# Women with fair phenotypes seem to confer a survival advantage in a low UV milieu. A nested matched case control study

**DOI:** 10.1371/journal.pone.0228582

**Published:** 2020-01-30

**Authors:** Pelle G. Lindqvist, Elisabeth Epstein, Mona Landin-Olsson, Måns Åkerlund, Håkan Olsson

**Affiliations:** 1 Department of Clinical Science and Education, Karolinska Institutet, Huddinge, Sweden; 2 Department of Obstetrics and Gynecology, Sodersjukhuset, Stockholm, Sweden; 3 Department of Endocrinology, Clinical Sciences, Lund University Hospital, Lund, Sweden; 4 Harvard Extension School, Harvard University, Cambridge, Massachusetts, United States of America; 5 Departments of Oncology and Cancer Epidemiology, Lund University Hospital, Lund, Sweden; Medical University of Gdańsk, POLAND

## Abstract

**Background:**

Sun exposure in combination with skin pigmentation is the main determinant for vitamin D status. Human skin color seems to be adapted and optimized for regional sun ultraviolet (UV) intensity. However, we do not know if fair, UV-sensitive skin is a survival advantage in regions with low UV radiation.

**Methods:**

A population-based nested case–control study of 29,518 Caucasian women, ages 25 to 64 years from Southern Sweden who responded to a questionnaire regarding risk-factors for malignant melanoma in 1990 and followed for 25 years. For each fair woman, defined as having red hair or freckles (n = 11,993), a control was randomly selected from all non-fair women from within the cohort of similar age, smoking habits, education, marital status, income, and comorbidity, i.e., 11,993 pairs. The main outcome was the difference in all-cause mortality between fair and non-fair women in a low UV milieu, defined as living in Sweden and having low-to-moderate sun exposure habits. Secondary outcomes were mortality by sun exposure, and among those non-overweight.

**Results:**

In a low UV milieu, fair women were at a significantly lower all-cause mortality risk as compared to non-fair women (log rank test *p* = 0.04) with an 8% lower all-cause mortality rate (hazard ratio [HR] = 0.92, 95% CI 0.84‒1.0), including a 59% greater risk of dying from skin cancer among fair women (HR 1.59, 95% CI 1.26‒2.0). Thus, it seem that the beneficial health effect from low skin coloration outweigh the risk of skin cancer at high latitudes.

**Conclusion:**

In a region with low UV milieu, evolution seems to improve all-cause survival by selecting a fair skin phenotype, i.e., comprising fair women with a survival advantage.

## Introduction

More pigmented skin, low sun light exposure, obesity and a life on high latitudes are risk factors for low vitamin D levels [1]. Human skin color seems to be adapted and optimized for regional sun ultraviolet (UV) intensity [2]. In regions close to the equator dense pigmentation seems to be selected by nature. At higher latitudes with less regional UV radiation, fair UV-sensitive skin is more common [3]. The fairer the skin, the shorter sun exposure is necessary for vitamin D production and other health effects [1, 4]. Evolution normally acts according to the principle of survival of the fittest. Thus, it is not unlikely that evolutionary forces are in effect regarding skin coloration. However, there is still no proof that fair skin provides an advantage in low UV milieu [5]. On the contrary, hundreds of papers have reported the positive relationship between fair skin and skin cancer. Thus, while skin cancer is the cost of fair skin, there might be other benefits. A fair phenotype is determined by several genes [6]. Red hair, for example, appears due to variations in the melanocortin 1 receptor (MC1R) gene [7]. Fitzpatrick type 1 is the palest skin type that always burns and never tans. It is associated with red hair and freckled skin. As a proxy for type 1 skin, we define women with red hair or freckles as having fair, UV sensitive, skin and all women not having red hair or freckles as non-fair skin.

The large Melanoma in Southern Sweden (MISS) cohort was designed to determine risk factors for malignant melanoma (MM). However, the cohort is also well-suited for an evaluation of possible beneficial effects of fair skin. For example, it gives us data on red hair color and freckles, along with detailed information on possible confounding factors, as well as follow-up regarding skin cancer and vital status. Southern Sweden is situated at 55^o^ N latitude, like southern Alaska. For 8 or 9 months of the year, the UV index remain under 3 (= low UV index); during late spring and summer it might rise to moderate levels at midday (3 to < 6); a few times a year it climbs to high levels (≥ 6) [8]. The prevalence of low vitamin D levels, a biomarker of the scarcity of UV radiation, is high. For example, a large part of all pregnant women in Southern Sweden were shown to have low vitamin D levels, i.e., 25-OH vitamin D < 50 nmol/L, especially during the winter [9, 10]. A Danish study showed individuals who had low-to-moderate sun exposure habits exhibited low vitamin D levels most of the year [11]. As opposed to fair-skinned individuals, women with dense skin pigmentation need a longer time in the sun in order to achieve sufficient levels of vitamin D, something that is not always possible [12]. If the above hypothesis of survival advantage is correct, fair women may have a survival advantage if they live in a low UV milieu. Our aim was to assess whether fair skinned women have a survival advantage to non-fair skinned in a low UV setting.

## Methods

In 1990 a cohort of approximately 39,973 Sweden born women (1000 per age from 25 and 64 years, with no history of malignancy, was drawn by random computerized selection from the general population registry of the South Swedish Health Care Region (Skåne, Blekinge and Småland). All women were born before 1966, i.e., before the large immigration started. Over 95% of women are Swedish born white Caucasians. The few non-Swedish born are mainly immigrants from Northern and East Europe. The cohort was followed up until 1 January 2016 and it represents one‒fifth of the entire regional South Swedish female population in the selected age-groups. The women were initially invited by letter to complete a standardized written questionnaire concerning risk factors for MM. That questionnaire administrated from 1990 to 1992 and resulted in 29,518 women entering in the study (response rate = 74%). The questionnaire covered several factors of potential interest for MM and life expectancy, such as sun exposure habits, marital status, educational level, smoking behaviour, comorbidity, hair colour, and freckles. A total of 162 women emigrated during the study and were subsequently censored from the analysis. Information was collected on mean personalized family income between 1990 and 1993 by means of official income and taxation records at Statistics Sweden (additional details at http://www.scb.se/en_/). In 2000 a second questionnaire was sent out to the same women, including also questions regarding weight, height and exercise habits. Women who subjectively reported themselves as having red hair or freckles were classified as having fair skin; and those without as having non-fair skin. Eumelanin is the dark pigment in the skin, while pheomelanin give red hair and freckles its red tone [2]. In our comparison, fair women would have high level of pheomelanin and low, to very low, level of eumelanin. Some might, however, have a low ability of facultative tanning [2]. Thus, thus the study is a comparison between the palest women and those somewhat less pale, not including women with olive or more pigmented skin. All women in southern Sweden with low-to-moderate sun exposure were defined as living in a low UV milieu.

We aimed to identify those with low-to-moderate sun exposure habits. Four questions were used regarding sun exposure: 1) How often do you sunbathe during the summer? (never, 1−14 times, 15−30 times, > 30 times); 2) Do you sunbathe during the winter, such as on vacation in the mountains? (no; 1−3 days, 4−10 days; > 10 days); 3) Do you use tanning beds? (never; 1−3 times per year; 4−10 times per year; > 10 times per year); and 4) Do you go abroad on vacation to swim and sunbathe? (never; once every 1–2 years; once a year; 2 or more times per year). The replies from the four questions were dichotomized into yes/no in the analysis (i.e., no or never versus sometimes). Sun exposure habits were categorized into three groups: zero ‘yes‘ responses (= low sun exposure); ‘yes‘ responses to one or two questions (= moderate exposure); ‘yes‘ responses to three or four questions (= greatest exposure). The main outcome was difference in all-cause mortality between fair and non-fair skinned women living in a low UV milieu. Vital statistics were drawn from the population registry up to 1 January 2016.

Baseline smoking habits at were recorded as; smokers, or non‒smokers. As a measure of co-morbid illness at the inception of the study, we created a dummy variable called ‘comorbidity’ to identify women who had been treated with either antidiabetic (Anatomical Therapeutic Chemical Classification System [ATC] A:10) or anticoagulant (ATC B:01) drugs or medications for cardiovascular disease (ATC C:01–C:10) for more than 1 month.

### Statistics

Descriptive statistical analysis of the total study population was performed using cross-tabulation with a 95% confidence interval (CI). We performed a nested matched case-control study based on variables known to be related to death and to sun exposure habits [13]. For each of the 11,993 women with fair skin according to their answers 1990 within the cohort, we identified all non-fair women in the remaining cohort population with the same; 5 year age group, marital status (married/not married), smoking habits (smoker/non-smoker), disposable income (low, middle, or high), education level (≥ 12 years or not), and comorbidity (present or not present), see Table 1. Out of all potential matches, one was drawn by random computerized selection (programmed in Python, Python Software Foundation, Beaverton OR, USA), i.e., a one-to-one matched design. This pair was included in analysis and the process continued with the next fair woman until all possible matched pairs from the original cohort (n = 29,518) were found (11,993 pairs).

**Table 1:** Characteristics of women included in the nested matched case control study, matched on six variables at inception of study.

	Fair skin^#^		Non-fair skin		
	n = 11993	%	n = 11993	%	*p*
Womens matched characteristics*					
Education > 12 years	4 114	34.3%	4114	34.3%	
Married		9 399	78.4%	9399	78.4%
Smoking	4 587	38.2%	4587	38.2%	
Disposable income					
Low	2346	19.6%	2346	19.6%	
Moderate	3654	30.5%	3654	30.5%	
High	5993	50.0%	5993	50.0%	
Co-morbidity	1043	8.7%	1043	8.7%	
Age (years)*	44.6	11.7	44.7	11.8	
Sun exposure Habits**					0.04
Not active	584	4.9%	634	5.3%	
Moderate active***	6438	53.7%	6 521	54.4%	
Most active****	4971	41.4%	4 838	40.3%	
Skin Cancer (SC)^##^	392	3.3%	271	2.3%	<0.001
Non-melanoma (NMSC)	238	2.0%	154	1.3%	<0.001
Malignant melanoma (MM)	162	1.4%	123	1.0%	0.02

# Fair skin is defined as having red hair or freckles, and non-fair as not having red hair or freckles

*Age per 5-year interval was used for matching, but mean is presented

** Assessed by 4 questions; Do you sunbathe during the summertime? Do you sunbathe during during the summertime? Do you sunbathe during the winter, such as on vacation in the mountains?

Do you use tanning beds? and Do you go abroad on vacation to swim and sunbathe?).

*** moderate active = answering yes on one or two of the sun exposure questions

****Most active sun exposure answering yes to 3 to 4 question

## 14 women had both NMSC and MM.

The main outcome is shown in Kaplan-Meier estimations to indicate differences in all-cause mortality between fair and non-fair skinned women in a low UV milieu. Cox regression analysis was performed to estimate of the hazard ratio (HR) of death, with adjustments for sun exposure and physical exercise. Since the two groups were matched also on age, we utilized the inception of the study as the beginning of its time scale. Time from inception was then calculated from inclusion until death, emigration, or 1 January 2016, whichever occurred first. We also analysed cause-specific HR of skin cancer mortality with its 95% CI by skin type. In model 3 and 4 in Table 2, time variable was time from January 2000 until death, emigration, or 1 January 2016, i.e., from when we had access to BMI and exercise data.

**Table 2:** Hazard ratios (HR) for all-cause death among women included in the nested matched case-control study both from inception 1990 (model 1 and 2) and in model 3 and 4 also data from year 2000 regarding all-cause mortality.

				Model 1		Model 2		Model 3*		Model 4*	
	Women	Women		Low UV		Adjusted		Strat analysis		Adjusted for Sun	
	Alive	dead		milieu		sun exposure		non-overweight		exp & exercise	
			%	HR	95% CI	HR	95% CI	HR	95% CI	HR	95% CI
Skin type											
Non-fair	10421	1572	13.1	1.0	Ref	1.0	Ref	1.0	Ref	1.0	Ref
Fair **	10498	1495	12.5	0.92	0.84–0.998	0.96	0.89–1.03	0.87	0.76–0.997	0.95	0.87–1.04
Sun exposure Habits											
low sun exp	813	405	33.3		Incl	1.0	Ref			1.0	Ref
Moderate sun exp^#^	11 097	1862	14.4		Incl	0.38	0.35–0.43			0.50	0.4–0.6
Greatest sun exp^##^	9 009	800	8.2		Not incl	0.21	0.19–0.24				0.32	0.3–0.4
Body mass index (BMI)											
< 25	10 014	869	8.0						Incl		
25-< 30	4 896	567	10.4						not incl		
≥ 30	1 721	288	14.3						not incl		
No answer	1 033	295	22.2						not incl		
Exercise habits*											
none	1 489	219	12.8							1.0	Ref
Moderate active	8 086	807	9.1							0.75	0.6–0.9
strenious	6 105	391	6.0							0.53	0.5–0.6
No answer	1 984	602	23.3							1.84	1.6–2.1

Incl = Included, Exp = exposure, Low UV milieu = having low-to-moderate sun exposure habits

* Data from women still in the study at second questionnaire year 2000.

** Fair skin was defined as having red hair or freckles

# answering yes on one or two of the sun exposure questions.

## answering yes om three or four of the sun exposure questions

**Fig 1:**
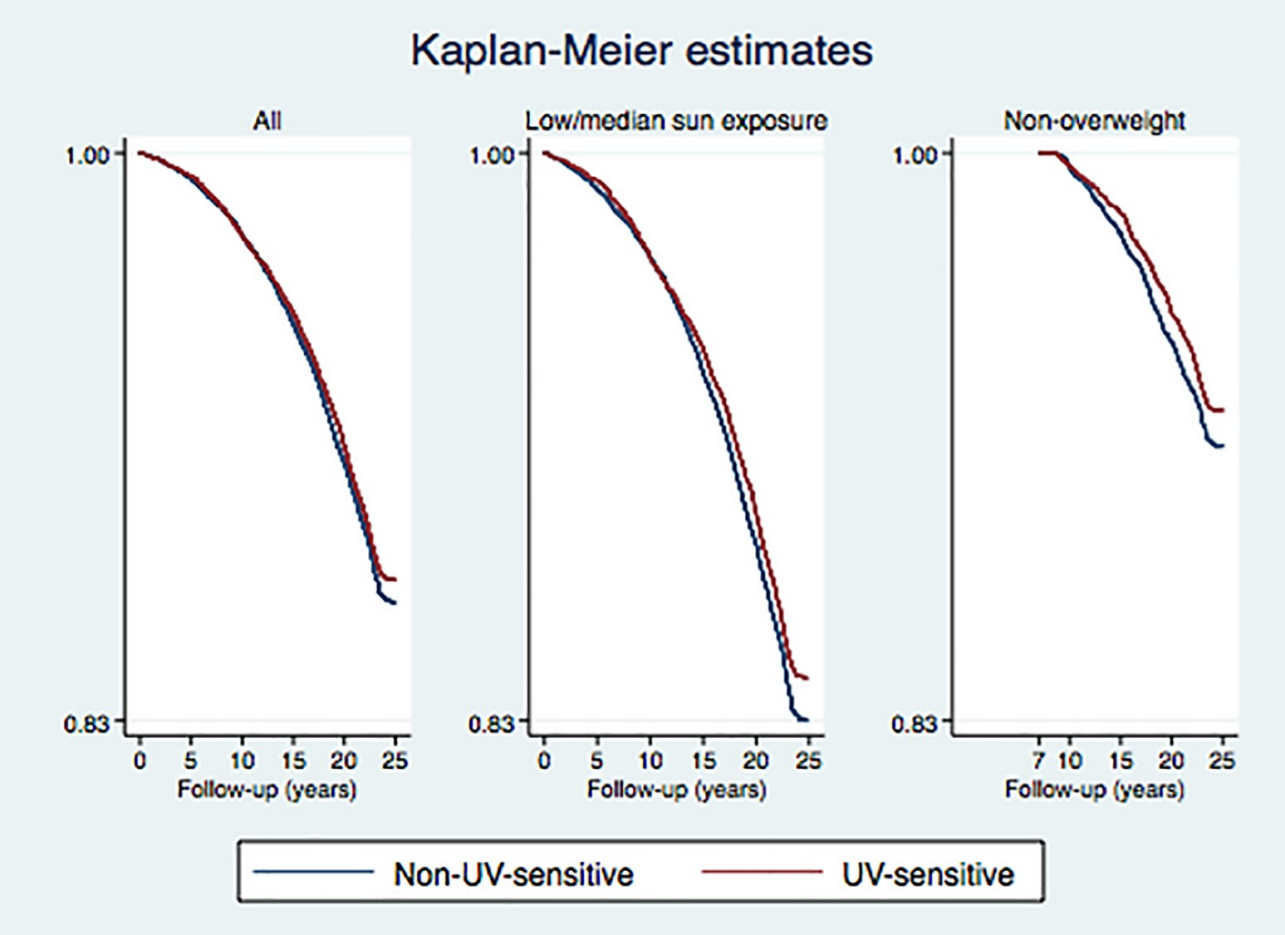
Kaplan-Meier (KM) graph of all-cause mortality in the total nested randomized observational study depending on UV‒sensitivity, difference log-rank test *p* = 0.14. a. Fig 1B. KM graph showing main outcome among low and moderate sun exposure groups log-rank test *p* = 0.044. Fig 1C KM graph left truncated follow-up among non-overweight women from year 2000. Log-rank test *p* = 0.045.

As a complement to the relative risk measures above, we also estimated the loss in mean life expectancy over a 25-year study period by quantifying the differences in restricted mean survival (RMS), i.e., the area under the survival curve between two time points. This provides a measure of mean survival among groups. We predicted the RMS based on a flexible parametric survival model that used restricted cubic splines to model baseline hazard function [14]. Specifically, we calculated the difference in RMS between fair and non-fair skinned women over a 25-year follow-up period in the nested matched case control study by age groups for those < 45, 45–54, and ≥ 55 years of age at inception, i.e., we collapsed the lower two age-groups due to low mortality rate. Further, we estimated the RMS using sun exposure and age groups with the greatest sun exposure groups as reference. IBM SPSS 23 software (Statistical Package for the Social Sciences, SPSS Inc., Chicago IL, USA) was used for descriptive and survival analysis. Python was used for programming the random maching, and Stata 13 (Statacorp, College Station TX, USA) for Fig 1. *P* values < 0.05 were considered statistically significant. The MISS study was approved by the Ethics Committee of Lund University (LU 632–03) and all women gave written consent for the data to be used for research.

## Results

There were 11,993 matched 1:1 pairs in the nested matched case-control study. Thus, a total of 23,996 out of 29,518 women formed the study population (Table 1). In the total study population there was no significant difference in all-cause mortality between fair and non-fair women (Fig 1A, *p* = 0.14).

Fair-skinned women in a low UV milieu (low-to-moderate sun exposure) were at a significantly lower all-cause mortality as compared to non-fair women (log rank test *p* = 0.044, Fig 1B), and at an 8% lower all-cause mortality rate (HR = 0.92, 95% CI 0.84–0.998) in cox regression (Table 2, model 1).

**Fig 2:**
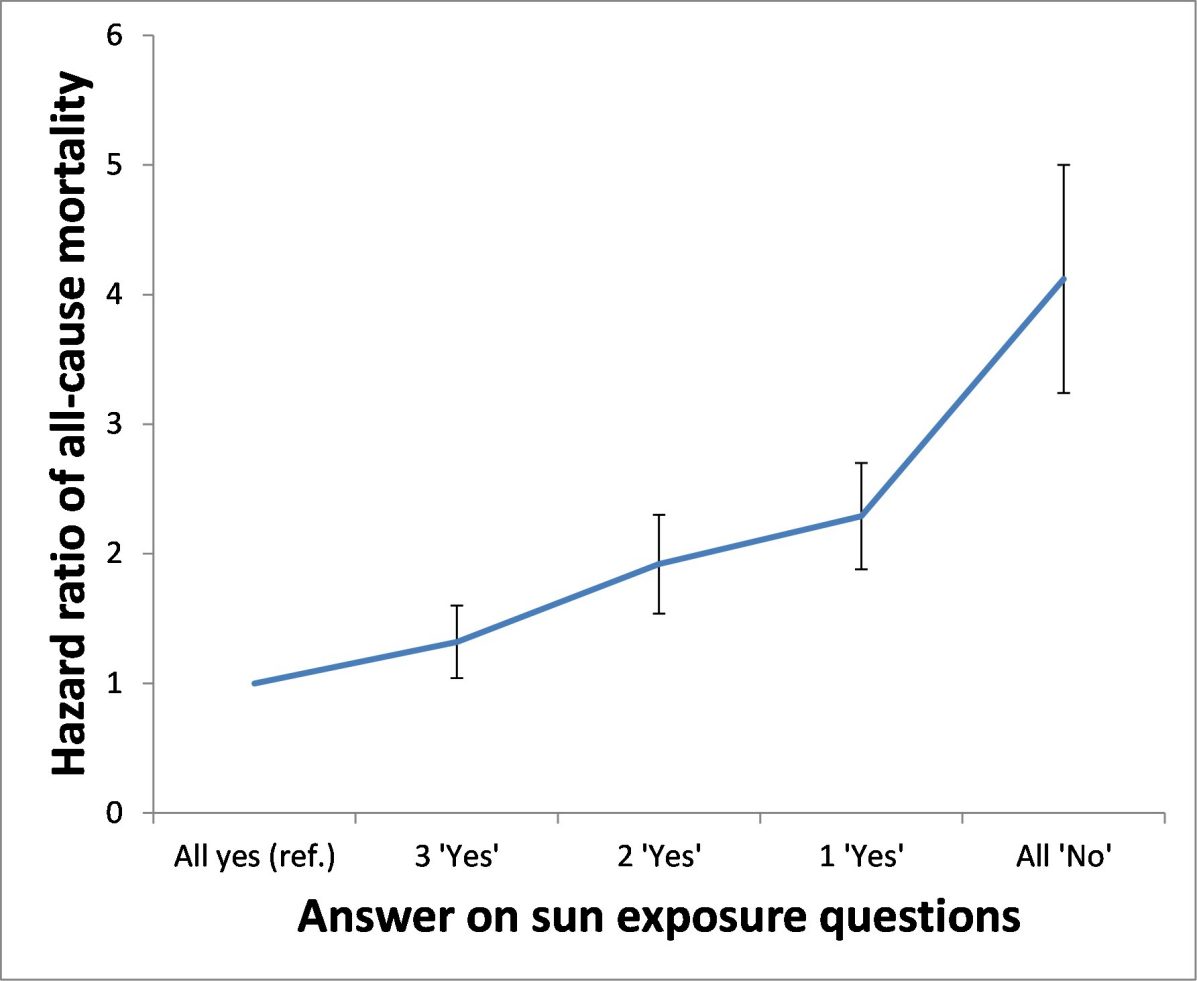
Sun exposure habits were classified by the number of “yes” answers to the following questions: (i) Do you sunbathe during the summer? (ii) Do you sunbathe during the winter, such as during holidays to the mountains? (iii) Do you use tanning beds? (iv) Do you go abroad on holiday to swim and sunbathe? Every “No” on the four sun exposure questions was related to ≥ 20% increased all-cause death rate in the next 25 years, as compared to those answering yes on more questions. Significance of difference between all five groups *p* = < 0.001, 95% confidence intervals are given.

In addition, the absolute differences in terms of RMS among non-fair women aged 45 to 54, and those above 54 at the inception had 1.2 months and 3.2 months shorter mean life expectancy during the ≈ 25-year study interval as compared to fair women. In comparing sun exposure by age groups (most active sun exposure = reference) in the total study population, the loss of mean survival clearly increased with increasing age and lower sun exposure habits. For example, as compared to women with greatest sun exposure and who were ≥ 55 years at inception, those in the same age group with moderate and low sun exposure habits had a mean life expectancy that was 23 and 27 months shorter, respectively, during the 25 year follow-up period, i.e., a difference of approximately one month per year shorter life-expectancy. Fig 2 shows the effect of all-cause mortality in the total study group by the number of “no” responses on the four sun exposure questions (“all yes” = reference) adjusted for all matched variables (all-yes HR = 1.0, three “yes” HR = 1.32 (1.1‒1.6), two “yes” HR = 1.92 (1.6‒2.3), one “yes” HR = 2.29 (1.9‒2.7), and all “no” HR = 4.12 (3.4‒5.0). All groups differed significantly (*p* < 0.001 between all groups).

Table 2 also shows the characteristics of the study population from year 2000 including access to information regarding body mass index (BMI) and exercise habits. In a stratified analysis of all non-overweight women (model 3), fair as compared to non-fair skinned women were at a significantly lower risk of death (log rank test *p* = 0.045). In a cox regression model there was a 13% lower all-cause mortality rate (HR = 0.87, 95% CI 0.76–0.997, Table 2, Fig 1C). HR of all-cause mortality in total cohort of fair women (model 4), after adjustments for sun exposure and exercise habits, was 0.95, (95% CI 0.87–1.04) (Table 2). Women with strenuous exercise habits were at half HR of all-cause mortality as compared to those inactive.

Fair women had a 59% higher mortality rate of skin cancer as compared to non-fair (HR = 1.59, 95% CI 1.26‒2.0). There were 392 and 271 cases of incidental skin cancer among fair and non-fair skinned women, respectively. The respective number of deaths among fair and non-fair skinned women with skin cancer during the study was 56 and 43, i.e., there were 13 extra deaths among fair women during the study interval. However, those extra deaths among fair women with skin cancer is “counterbalanced” by 90 fewer deaths related to other causes, resulting in 77 fewer deaths among fair women. The number of ovarian-, uterus-, cervical-, and colorectal cancer occurred in 67 vs. 57 (*p* = 0.4), 104 vs. 113 (*p* = 0.5), 21 vs. 24 (*p* = 0.7) and 48 vs. 56 (*p* = 0.4), among fair and non-fair women, respectively.

## Discussion

Women with a fair UV-sensitive phenotype living in a low UV milieu had a significantly increased life expectancy as compared to non-fair women. Fair women were at an eight percent lower all-cause mortality rate, as compared to those with non-fair skin. There is a strong inverse dose-dependent risk between increasing sun-exposure habits and all-cause mortality.

### Strengths and limitations

Our large sample, comprising 20% of all women in the south Swedish region between 25 and 64 ages, as drawn by random selection from the population registry at the study inception 1990 is a strength. It was thus a representative sample of the South Swedish population at the time of recruitment before the large immigration of the 2000’s. It comprises almost exclusively European Caucasian women. Thus, the comparison between fair and non-fair was mainly a comparison between Fitzpatrick types 1 skin vs. type 2‒3 skin. Since the questionnaire was administrated at the inception of the study, there was no recall bias. Since we earlier have been criticized that our adjustments in Cox regression might not be adequate, we decided to perform a one-to-one matched design. Historically, during evolution there was no possibility to use solarium or to travel for sunbathing. Therefore, we were predetermined to make the main outcome comparison in a low UV milieu, i.e., among those with low-to-moderate sun exposure habits. As secondary outcome we assessed mortality by sun-exposure with adjustment for exercise or stratified for low BMI, only including the time period after year 2000. A major limitation is that the significance level of the lower risk of all-cause mortality among fair women was close to the 5% significance level in all analyses regarding skin type, but it was according to the predetermined hypothesis. Another strength is that the analyses from year 2000 including exercise habits, and BMI showed similar results, but with wider CIs. The results might not be generalized into regions with more intense UV radiation. The aim of the study was not to assess cause specific mortality. However, it is impossible to publish on beneficial effects by sun exposure without including data on skin cancer mortality. Thus, our study is in agreement with the large amount of papers showing an increased incidence of skin cancer with fair skin and we also showed increased mortality in skin cancer. Since fair skin is selected at high latitudes, an improved all-cause survival is also expected from an evolutionary perspective [2]. Frost and coworkers reported in an open internet-based study that red-haired women were particularly prone to ovarian-, uterus-, cervical, and colorectal cancer, our results could not reproduce these findings and we did not find an increased incidence of these groups among fair women in our study [15]. There has been somewhat conflicting evidence regarding sun exposure and all-cause mortality. The Swedish Women´s Lifestyle and Health Study reported that increased sun exposure (measured as sunbathing holidays, i.e., which was one of our four questions) was related to reduced HRs for all-cause mortality [16]. On the other hand, a large US epidemiological study based on regional, not personal, UV radiation reported a positive relation between increasing UV radiation and all-cause mortality [17]. A possible explanation for the opposing results might be the differences in latitude and, therefore, UV intensity (Sweden latitude 55^o^ to 59^o^ and continental US latitude 24^o^ to 42^o^. While the mean level of the biomarker vitamin D for sun exposure was 48.6 (± 20.5) nmol/L in Sweden it was 77.0 (± 25.0) nmol/L in the US, indicating a greater problem with sun deficiency at high latitudes [9, 18]. Based on data from the Swedish Meteorological and Hydrological Institute (SMHI), in 2014 there was one day with strong UV exposure, i.e., UV-index ≥ 6.

### Skin cancer mortality

When we investigated whether the increased mortality associated with skin cancer influenced the strong inverse relationship between all-cause mortality and increasing sun exposure habits and found that this was not the case. Women with fair skin were at a 59% increased risk of death in skin cancer. This was counterbalanced by the health benefits, as measured by all-cause mortality, of fair skin and sun exposure. There is an increased risk of skin cancer with both fair skin and increasing sun exposure, but the prognosis of skin cancer seem to improve with increasing sun exposure [19, 20]. Thus, there seem to be a tradeoff between health benefits and skin cancer and in regions with scarcity of solar UV radiation fair skin have been selected [2]. In our modern society there is not unusual with a mismatch between skin coloration and geography/climate/ habits that might cause increased morbidity and mortality [2].

### Sun exposure and overweight

Overweight and obese women do not seem to obtain the same benefit from having fair skin or from sun exposure as non-overweight women. We have seen similar findings in prior studies, where the lower risk of type 2 diabetes mellitus and endometrial cancer after UV exposure was mainly seen in non-overweight women [21, 22]. Wortsman and coworkers have clearly demonstrated that obesity has a detrimental effect on vitamin D levels for a given amount of UV exposure [23]. Thus, lower sun exposure habits among overweight is not the cause. It appears that vitamin D is either produced in a smaller quantity or consumed/inactivated among overweight women. Further, a study using Mendelian randomization analysis showed that increasing BMI leads to lower vitamin D levels [24]. The differential impact of BMI by sun exposure on all-cause mortality is an area that would benefit from additional research. Since BMI seem to be in the causal pathway of sun exposure and all-cause mortality, we chose not to adjust for BMI and present only stratified analysis.

It has been hypothesized that the inbreeding with Neanderthals some 47,000 to 65,000 years ago in northern Canaan might have helped Homo sapiens adjust to life beyond Africa [25–27]. Studies of the ancient Neanderthal genome have shown that Westerners carry approximately 1% to 3% of Neanderthal DNA [25, 26]. People of European origin are highly likely (≈ 60% to 70%) to have the Neanderthal DNA that affects keratin filaments, i.e., zinc finger protein basonuclin-2 (BNC2). The latter alleles are thought to be involved in the adaptive variation of skin coloration, influencing skin pigmentation towards fairer skin [6, 28]. With our finding of increased life expectancy with fair skin, we speculate that the preserved high carriership of the Neanderthal BNC2 allel might be an advantage at high latitudes.

We interpret our findings to support that a fair, UV-sensitive phenotype in Sweden seems to be related to prolonged life expectancy in a low UV milieu, but at the cost of an increased risk of death due to skin cancer. Over thousands of years a fair UV-sensitive phenotype has possibly been selected for optimal health at high latitudes.

## Supporting information

S1 File (DBF)
